# Isolated Fallopian Tube Torsion

**DOI:** 10.1155/2013/479698

**Published:** 2013-07-30

**Authors:** S. Kardakis, A. Barranca, A. Vitelli, I. Amore, F. Trento, G. Caccia

**Affiliations:** Department of Obstetrics and Gynecology, Regional Hospital OBV, Via Turconi 23, Ticino, 6850 Mendrisio, Switzerland

## Abstract

Isolated torsion of the Fallopian tube is a rare gynecological cause of acute lower abdominal pain, and diagnosis is difficult. There are no pathognomonic symptoms; clinical, imaging, or laboratory findings. A preoperative ultrasound showing tubular adnexal masses of heterogeneous echogenicity with cystic component is often present. Diagnosis can rarely be made before operation, and laparoscopy is necessary to establish the diagnosis. Unfortunately, surgery often is performed too late for tube conservation. Isolated Fallopian tube torsion should be suspected in case of acute pelvic pain, and prompt intervention is necessary.

## 1. Introduction

Torsion of the fallopian tube without ovarian torsion is a rare cause of lower abdominal pain in reproductive-age women with an incidence of 1 in 1.5 million women [1].

Preoperative diagnosis is difficult often leading to delay of timely intervention. Prompt surgical intervention is necessary to establish the diagnosis and for an adequate treatment. We report a case of isolated tubal torsion in a patient with hydrosalpinx and history of Chlamydia infection.

## 2. Case Presentation

A 38-year-old, gravida 0 woman presented to the gynecological emergency room with complaint of severe, constant low abdominal pain (VAS 8). She denied any radiation of pain, fever, chills, nausea, or vomiting. No urinary symptoms, vaginal discharge, or bleeding were presented. She was in middle cycle and was not taking any contraceptive. She reported dysmenorrhea and painful intercourse the last year such as Chlamydia infection ten years earlier. 

Three days before, the patient experienced sudden onset of severe, right lower quadrant pain and presented to the emergency surgical department where she was treated for suspect renal colic and urine infection. The pain recurred the next day and became continued. One year ago, the patient was admitted to surgical department for renal colic and during her hospitalization a CT scan was performed and showed an elongated cystic mass on the right adnexa. 

On physical examination, focal tenderness was presented in the right lower quadrant. On pelvic examination, right ovarian tenderness was noted and there was no cervical motion tenderness. Complete blood count and urinalysis were normal, CRP was 47, and urine pregnancy test was negative. Endocervical swabs were obtained and were negative. 

Sonography examination showed a normal uterus and a normal left ovary. A small amount of simple free fluid was present within the Douglas pouch. A cystic mass measuring 8 × 4 × 3.5 cm was thought to arise from the right adnexa or ovary. Carefully examination demonstrated that the tubular cystic structure was separated from the ovary and was presenting two ecographic features: the first one was a fusiform, thick-walled cystic mass resembling a hydrosalpinx and the second one had the appearance of a cyst containing blood. Differential diagnosis included isolated torsion of Fallopian tube, paraovarian cyst, hydrosalpinx associated with tube-ovarian abscess, or endometrioma. CT examination did not add useful information; the mass was similar at the image already presented in the previous CT. 

**Figure 1:**
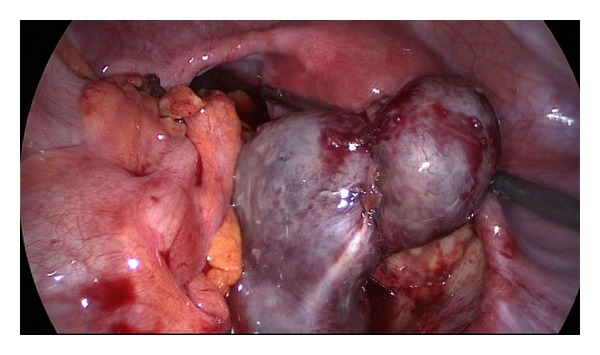
Fallopian tube torsion.

**Figure 2:**
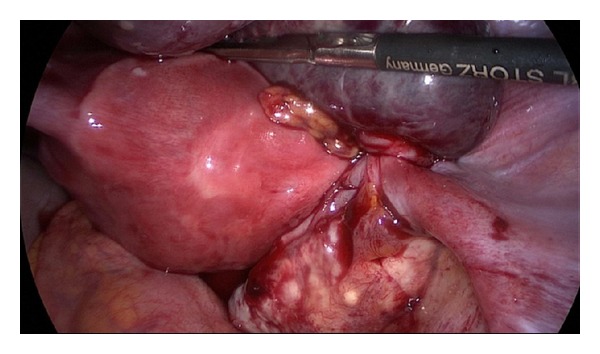
Fallopian tube torsion.

**Figure 3:**
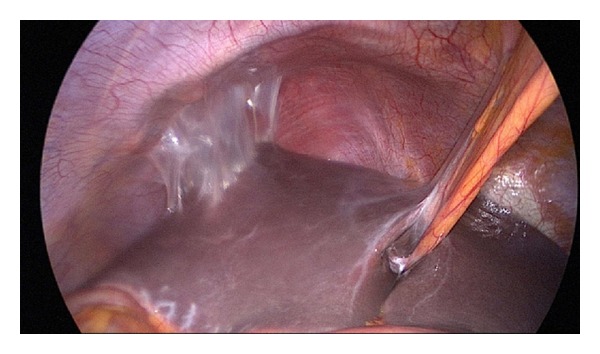
Fitzhung-Curtis syndrome.

An empiric antibiotic therapy was initiated and the patient underwent laparoscopy. The left ovary and tube were adherent to the pelvic wall. Uterus had a normal appearance. On the right adnexa, the tube was dilated, twisted about its longitudinal axis and necrotic (Figures 1 and 2). The ovary was normal in appearance. Omental adhesions were presented in the right lower quadrant and characteristic feature of “violin string” adhesions that extended from the anterior surface of the liver to the peritoneum suggested a Fitzhugh-Curtis syndrome (Figure 3). A right salpingectomy was performed. Cultures from the peritoneal fluid were obtained and were negative. Finally the diagnosis of tube torsion and hydrosalpinx was confirmed by histopathological examination. 

## 3. Discussion

Isolated tubal torsion is a rare event and the exact mechanisms that lead to torsion of the fallopian tube are not well understood. Some intrinsic and extrinsic factors have been documented. Intrinsic causes include hydrosalpinx, haematosalpinx, tubal neoplasms, prior surgery such as tubal ligation, physiological abnormalities such as hypermotility, tubal spasms and abnormal peristalsis, congenital abnormalities, such as incomplete distal mesosalpinx, excessive length, or spiral course of the tube, and hydatids of morgagni. Extrinsic factors include ovarian or paraovarium mass, tubal adhesions, uterine enlargement due to pregnancy or tumor, the Sellheim theory relating to sudden body position changes, trauma, and venous congestion in the mesosalpinx [2, 3]. Our patient had hydrosalpinx and pelvic adhesions, presumable after previous PID. The history of pelvic pain the last year suggests a state of chronicity. Undiagnosed torsion may undergo alternative states of mild torsion-detorsion that finally bring the condition to chronicity.

The clinical presentation of ovarian torsion is nonspecific and therefore is a challenge for the clinician to recognize and differentiate from multiple other etiologies The preoperative diagnosis of tubal torsion is difficult because of no pathognomonic symptoms and clinical findings. Acute severe lower abdominal pain is always present and often in the per ovulatory period probably because of pelvic congestion and increased tubal motility at mid cycle. The pain can be constant and dull or paroxysmal and sharp, radiating to the thigh or groin. Nausea and vomiting may accompany the pain. On clinical exam, findings include abdominal tenderness with or without peritoneal signs. On pelvic exam adnexa tenderness is present but a mass is not always palpable. Laboratory findings are usually nonspecific. Necrosis can cause leukocytosis. The sedimentation rate or CRP can be elevated. Occasionally, the patient may have fever [4]. 

Differential diagnosis can involve ectopic pregnancy, endometriosis, pelvic inflammatory disease, ovarian torsion, ruptured ovarian cyst, degenerative leiomyoma, acute appendicitis, and other gastrointestinal and urinary conditions [5]. Isolated tubal torsion is uncommon; however, it is important to be considered in the differential diagnosis of acute lower abdominal pain because a delay of intervention may result in failure to save the tube [4].

Many reports suggest that tube torsion predominantly affects women in the reproductive age and more on the right side, possibly because of partial immobilization of the left tube by its proximity to the sigmoid mesentery on the left side and the relatively less venous flow on the right side. In addition, it is more likely to operate patients with right-sided lower abdominal pain because of suspicion for appendicitis [5].

Initially the mechanical obstruction of adnexal veins and lymphatics with blood flow in the afferent arteries relatively unchanged results in pelvic congestion, edema and enlargement of the fimbrial end, and subsequent partial to complete torsion of the involved tube [6]. Complications include tube necrosis and gangrenous transformation, leading to superinfection and peritonitis [7]. Local necrosis can also result in irreversible damage to the ipsilateral ovary [8].

Although rare, it is important to recognize the possibility of this diagnosis in the setting of hydrosalpinx with a sonographically normal ovary in a patient with acute pain, as delay in diagnosis and treatment may result in increased morbidity. The most consistent finding on either CT or ultrasound is a midline cystic mass, either in the posterior cul-de-sac or superior to the uterus, associated with a normal ipsilateral ovary [9]. Origoni et al. [10] suggest the use of Doppler flow ultrasound technique to make a differential diagnosis in case of total adnexal torsion. An isolated tubal torsion should be considered when a detailed Doppler flow ultrasound shows a normal ovary and a pelvic cyst.

The diagnosis is generally made at time of surgical exploration. Prompt consideration of this diagnosis and surgical detorsion may prevent irreversible vascular changes [11].
